# Difference in Yield and Physiological Features in Response to Drought and Salinity Combined Stress during Anthesis in Tibetan Wild and Cultivated Barleys 

**DOI:** 10.1371/journal.pone.0077869

**Published:** 2013-10-24

**Authors:** Imrul Mosaddek Ahmed, Fangbin Cao, Mian Zhang, Xianhong Chen, Guoping Zhang, Feibo Wu

**Affiliations:** Department of Agronomy, College of Agriculture and Biotechnology, Zijingang Campus, Zhejiang University, Hangzhou, China; Universidad Miguel Hernández de Elche, Spain

## Abstract

Soil salinity and drought are the two most common and frequently co-occurring abiotic stresses constraining crop growth and productivity. Greenhouse pot experiments were conducted to investigate the tolerance potential and mechanisms of Tibetan wild barley genotypes (XZ5, drought-tolerant; XZ16, salinity/aluminum tolerant) during anthesis compared with salinity-tolerant *cv* CM72 in response to separate and combined stresses (D+S) of drought (4% soil moisture, D) and salinity (S). Under salinity stress alone, plants had higher Na^+^ concentrations in leaves than in roots and stems. Importantly, XZ5 and XZ16 had substantially increased leaf K^+^ concentrations; XZ16 was more efficient in restricting Na^+^ loading in leaf and maintained a lower leaf Na^+^/K^+^ ratio. Moreover, a signiﬁcant decrease in cell membrane stability index (CMSI) and an increase in malondialdehyde (MDA) were accompanied by a dramatic decrease in total biomass under D+S treatment. We demonstrated that glycine-betaine and soluble sugars increased signiﬁcantly in XZ5 and XZ16 under all stress conditions, along with increases in protease activity and soluble protein contents. Significant increases were seen in reduced ascorbate (ASA) and reduced glutathione (GSH) contents, and in activities of H^+^K^+^-, Na^+^K^+^-, Ca^++^Mg^++^-, total- ATPase, and antioxidant enzymes under D+S treatment in XZ5 and XZ16 compared to CM72. Compared with control, all stress treatments significantly reduced grain yield and 1000-grain weight; however, XZ5 and XZ16 were less affected than CM72. Our results suggest that high tolerance to D+S stress in XZ5 and XZ16 is closely related to the lower Na^+^/K^+^ ratio, and enhanced glycine-betaine and soluble protein and sugar contents, improved protease, ATPase activities and antioxidative capacity for scavenging reactive oxygen species during anthesis. These results may provide novel insight into the potential responses associated with increasing D+S stress in wild barley genotypes.

## Introduction

Drought and salinity are the most important abiotic stresses limiting agricultural production worldwide. These two abiotic stresses frequently co-occur in both natural and agricultural ecosystems as salinity becomes concentrated in the remaining soil solution especially under drought condition [1,2]. Therefore, an improvement in drought and salinity tolerance in crops is a pre-requisite for achieving greater economic gains. The best and most effective approach in this regard is to develop drought- and salinity- tolerant crop varieties. It is therefore important to identify the genetic resources that have high tolerances and to understand the mechanisms of drought and salinity tolerance in plants. 

Remarkable studies have been done concerning drought and salinity tolerance separately in cultivated crops, and considerable advances have been made in the development of crop varieties tolerant to drought or salinity [3-9]. The identification of well-adapted wild relatives (e.g. annual wild barley, *H. spontaneum* C. Coch for barley) that are able to grow well in drought and/or saline soils also provides a useful supply of new germplasm for future breeding [10]. It is also important to investigate the physiology of drought and salinity tolerance in well-adapted wild barley, to understand the limits and tradeoffs between drought and salinity tolerance, and to determine the traits that are associated with high tolerance to both factors [11,12]. In our previous study, we successfully identified Tibetan wild annual barley genotypes XZ5 and XZ16 that exhibited high tolerance to drought and salinity stress, respectively [13,14]. The underlying physiological and biochemical mechanisms involved in drought and salinity tolerance remain unclear, thus preventing the optimization of gene identification techniques and its further commercial applications. 

Plants usually share a common response to salinity and drought stress. Water deficits or osmotic effects are most likely the major physiological mechanisms that cause growth reduction as both stresses reduce the water potential in soil. In addition to the toxic effects of sodium and chloride ions, salinity disturbs the water relations of plants due to a decreased availability of water in soil as a result of reduced osmotic potential [15]. It was assumed that low soil moisture (drought) in a saline soil would aggravate salt toxicity, further impeding root growth. In this scenario, the acquisition of water from the subsoil may be strongly restricted, along with the ability of the plants to withstand drought stress [16]. Therefore, a complete understanding of the combinational responses of plants to these two stresses and effects on plant growth is of considerable, practical and ecological significance for the improvement of abiotic stress tolerance.

Barley ranks the fourth among the cereal crops worldwide in planting area and production. Due to the rapid loss of genetic variation through cultivar replacement, modern barley cultivars have become more sensitive to abiotic and biotic stresses, and their monotonous genetic background has been a bottleneck in cultivar improvement. Wild barley germplasm is a treasure trove of useful genes and offers a rich source of genetic variation for use in crop improvement. Our recent studies have demonstrated that Tibetan wild barley (*H. vulgare* L. *ssp.* *spontaneum*) genotypes XZ5 and XZ16 have a high tolerance/resistance to drought and salinity stress, respectively, during the vegetative stage [17]. However, crop plants are especially sensitive to drought stress during the early reproductive stage [18,19]. Therefore, a question arises as to whether the Tibetan wild barley genotypes XZ5 and XZ16 are tolerant to combined stresses of drought and salinity at anthesis stage. If this is the case, the question arises whether the mechanisms of drought and salinity tolerance in these two Tibetan wild barley genotypes are different from those in cultivated barley. Thus, the main objective of the present study was to compare the morphogenetic and physiological effects of combined drought and salinity stresses on the wild and cultivated barley genotypes at anthesis. The study also improved our understanding of stress avoidance mechanisms that can be executed to enrich cultivated barley for multiple stress tolerance.

## Materials and Methods

### Plant materials and experimental design

A pot experiment was conducted in a greenhouse of Zijingang Campus, Zhejiang University, Hangzhou, China in 2011-2012. Agricultural soil was collected from an experimental farm (depth 0-15 cm) at Huajiachi Campus, Zhejiang University. The soil was air-dried and mixed daily until 8% water content was reached. Air-dried soil was sieved and plastic pots (5 L, 20 cm height) were filled with 4.5 kg of air-dried soil. The soil used in this study had a pH of 6.9. The total N content, available P and K contents were 2.4 g kg^-1^, 38.2 mg kg^-1^ and 31.5 mg kg^-1^, respectively. Soil textural analysis showed the following composition: sand 65.0%, silt 28.8% and clay 6.2%. This composition indicates that this soil can be classified as a silt loam. Each soil pot was fertilized with 1 L basal nutrient solution (BNS). The composition of the BNS was as described by Wu et al. [20]. 

Two Tibetan wild genotypes XZ5 and XZ16, were identified as drought-tolerant and salinity/Al-tolerant, respectively [13,14,21] and a salinity-tolerant *cv* CM72 [22] was also used in this study. Seeds were sown on 25^th^ of October 2011, directly in each pot one week after application of BNS. At 10 d after emergence, seedlings were thinned to 8 uniform plants per pot. Drought and salinity treatments were imposed during anthesis. In our experiments, we considered the beginning of pollination to be an anthesis period. At this stage, we manually opened the flower and observed the anther carefully. The appearance of yellow anthers inside the flower was the criteria used to select ‘anthesis’ period. We started drought and salinity stresses on all genotypes on 27 February 2012. This experiment included the following 4 treatments: (1) control (non-salinized), in which pots remained humid (at a 60-80% water holding capacity) throughout; (2) drought stress (D) treatment, in which 1 L water was added to each pot and the plants were then subjected to drought stress over 20 d by withholding irrigation until the soil moisture content (SMC) was reduced to 4%; (3) salinity stress treatment (S), in which 1 L of a 200 mM NaCl solution was added to each pot and the soil remained humid (at a 60-80% water holding capacity) throughout; and (4) combined drought and salinity stress (D+S) treatment, in which 1 L of a 200 mM NaCl solution was added to each pot and the plants were then subjected to drought stress by withholding irrigation until SMC reduced to around 4%. The experiment was arranged in a split-plot design, with stress treatments as the main plot and genotypes as the sub-plot with nine replicates. Electrical conductivity of the soil in salinity and D+S treatment was 5.20 ds m^-1^, as measured by EC_1:1_ soil water suspension method described by Rhoades et al. [23]. Soil moisture was measured daily using an HH2 Moisture Meter (Delta-T Devices, Cambridge, UK). 

At the end of drought stress treatment (SMC 4%), the flag leaves were sampled to measure physiological parameters. The plant samples were harvested and plant height, dry weight and Na^+^ and K^+^ concentrations were measured. All pots were then re-irrigated to maintain SMC of approximately 30-40% until the seeds were harvested. Grains were harvested at maturity, and yield and yield components were examined. 

### Growth Measurement and Na^+^ and K^+^ Concentration Analyses

At the end of drought stress treatment (SMC 4%), four replicates of plants were gently uprooted and rinsed thoroughly with running tap water. After measuring plant height, plants were separated into roots and shoots (stems and leaves). The roots and shoots were dried at 75 °C for 72 hours to a constant weight. The dried samples were then weighed, powdered, and again weighed before ashing at 550 °C for 12 h. The ash was digested with 5 mL of 30% HNO_3_ and diluted with deionized water [24]. The concentrations of Na^+^ and K^+^ were determined by flame atomic absorption spectrometry (Shimadzu, AA-6300, Kyoto, Japan).

### Measurement of chlorophyll content and photosynthetic parameters

Chlorophyll content and photosynthetic parameters were measured on the flag leaf using 5 replicates. Chlorophyll a (Chl a), chlorophyll b (Chl b) and total carotenoids contents were determined according to Arnon [25]. A LI-6400 portable photosynthesis system (LI-COR, Lincoln, NE) was used to measure net photosynthetic rate (Pn), stomatal conductance (*gs*), transpiration rate (Tr) and intracellular CO_2_ concentration (Ci) of in flag leaves. 

### Determination of soluble protein, glycine-betaine (GB) and soluble sugar contents and protease activity

Soluble protein content was measured according to Bradford [26] using bovine serum albumin as a standard. The protease activity was determined using the casein digestion assay described by Drapeau [27]. One unit equals the amount of enzyme needed to release acid soluble fragments equivalent to 0.001 A280 per minute at 37 °C and pH 7.8. The glycine-betaine content was estimated in dried leaf powder according to Greive and Grattan [28]. Soluble sugars were estimated using anthrone reagent method [29].

### Determination of lipid per oxidation and cell membrane stability index and assay of antioxidant enzyme activities

The fresh flag leaves were sampled and immediately put in liquid nitrogen for measurement of antioxidant activity and other variables. Leaf samples (0.3 g) were then homogenized in 8 mL 50 mM phosphate buffer (PBS, pH 7.8) using a pre-chilled mortar and pestle. The homogenates were then centrifuged at 10,000 *g* for 15 min at 4 °C and the supernatants were used in an assay to determine the malondialdehyde (MDA) content and antioxidant enzyme activity. The MDA content and the superoxide dismutase (SOD, EC 1.15.1.1), catalase (CAT, EC 1.11.1.6) and peroxidase (POD, EC 1.11.1.7) activities were determined according to Wu et al. [20]. Ascorbate peroxidase (APX, EC 1.11.1.11) activity was determined according to Chen et al. [30]. Cell membrane stability index (CMSI) was determined by estimating ion leaching from leaf tissue into distilled water according to the method described by Huang et al. [31].

### Determination of ASA and GSH contents and ATPase activity

The ASA content was determined according to the method described by Law et al. [32]. Reduced glutathione (GSH) activity was determined using a GSH colorimetric activity assay kit (Jiancheng Bio Co., Nanjing, China). Activities of H^+^K^+^-, Na^+^K^+^-, Ca^++^Mg^++^- and total-ATPase were determined by measuring the release of Pi using an activity assay kit (Jiancheng Bio Co., Nanjing, China; http://www.njjcbio.com/html/search.php) [33].

### Determination of total phenolic (TP) content

Total phenolic (TP) content was determined using the Folin-Ciocalteu reagent, described by Singleton et al. [34] with minor modifications. A 0.5 g sample of dried flag leaves was macerated and extracted in 6 mL of 80% (v/v) methanol and thoroughly shaken at room temperature for 1 h, followed by centrifugation at 3500 rpm for 10 min. An aliquot of 1/10 diluted supernatant was oxidized using Folin-Ciocalteu’s phenol reagent (Sigma-Aldrich, St. Louis, MO, USA). The reaction was neutralized using 7.5% (w/v) Na_2_CO_3_. The samples were vortexed and incubated for 2 h at room temperature in dark. Absorbance was measured at 765 nm using a Lambda 35 UV/Vis spectrophotometer (PerkinElmer Ltd., Shelton, CT, USA). The total phenol content was quantiﬁed by external calibration using gallic acid (Sigma-Aldrich, St. Louis, MO, USA) as a standard. The samples were independently analyzed in triplicate and results were expressed as milligram of gallic acid equivalents (GAE) per gram of extract (mg GAE g^-1^).

### Measurement of yield and yield components

Spike lengths, grains per spike, 1000-grain weight and grain yield per plant were counted at maturity stage. Spike length was measured as the length from neck node to tip of the upper most spikelet. 

### Statistic analysis

All data were presented as mean values for each treatment. An analysis of variance was conducted between different treatments. The signiﬁcance of the differences between Tibetan wild and cultivated barley or between control and research treatments were evaluated by LSD multiple range tests (P <0.05) using the SAS 9.2 (SAS Institute Inc., Cary, NC, USA) statistical software. Correlation analysis was performed to determine the relationship between plant ion concentrations and relative plant dry weight using SAS CORR procedure. Origin Pro version 8.0 (Origin lab corporation, Wellesley Hills, Wellesley, MA, USA) was used to prepare graphs.

## Results

### Plant height and biomass accumulation

Compared with control, plant height was apparently reduced by drought and salinity either alone, or in combination. Significant genotypic differences were seen as follows: decreased by 14.2%, 6.3%, 20.6% in XZ5; 18.4%, 10.4%, 28.1% in XZ16 and 31.1%, 17.7%, 36.3% in CM72 under drought, salinity, D+S treatments, respectively (Table S1). Meanwhile, plant dry weight under drought, salinity, D+S treatments decreased by 12.6%, 15.5%, 26.6% in XZ5; 10.0%, 14.3%, 20.8% in XZ16 and 16.8%, 16.1%, 28.2% in CM72, respectively. 

### Yield and Yield Components

**Figure 1:**
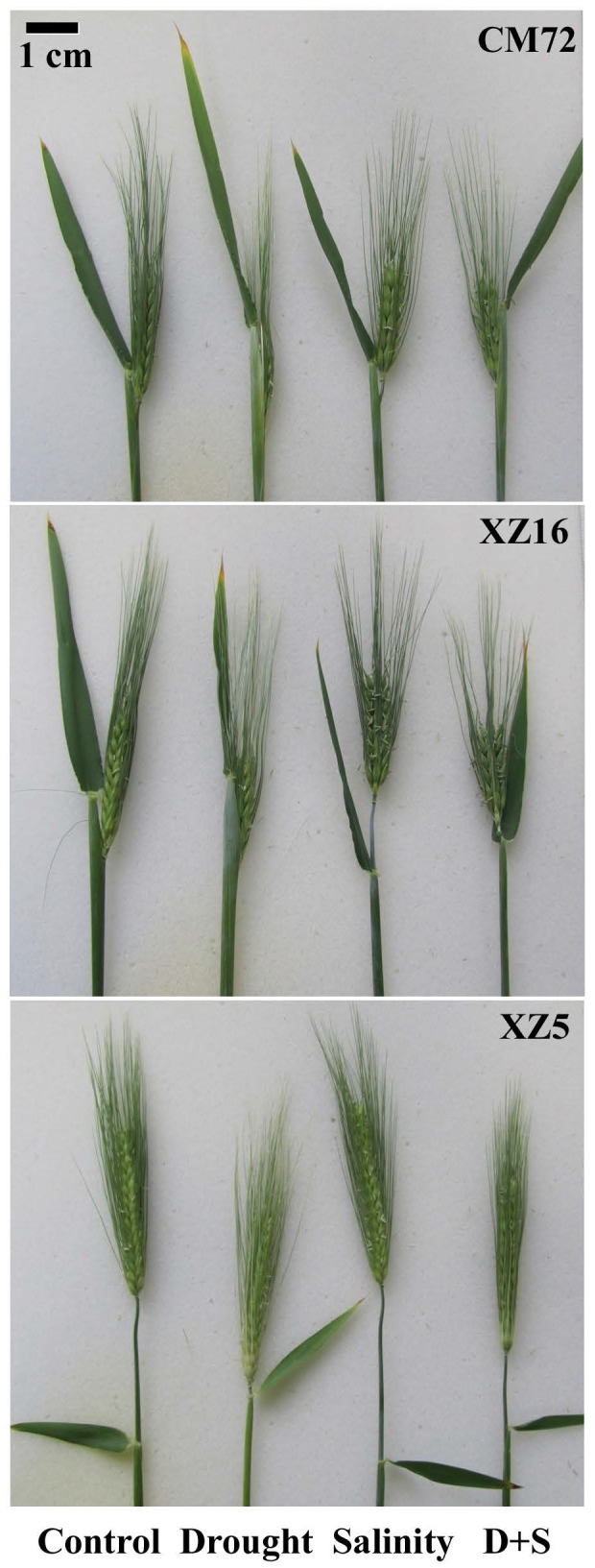
Phenotype of spikes of cultivated and Tibetan wild barley genotypes. Plants were affected by drought, salinity alone and combined stresses (D+S) during anthesis stage at 4% soil moisture level.

The phenotypic changes in spikes of XZ5, XZ16 and CM72 during drought and salinity stresses either alone or in combination are shown in Figure 1. Spike growth significantly differed among the genotypes under drought and D+S treatments during anthesis stage at a soil moisture level of 4%. Heading was inhibited in XZ16 and CM72 under drought and D+S treatment, which was not seen in XZ5. Under drought stress, spike fully emerged from the sheath in XZ5; spikes of CM72 were nearly circled by flag leaf sheath and 50% of spikes in XZ16 emerged clearly. 

Compared with control, spike length significantly (P<0.05) decreased in CM72 and XZ16 under all stress conditions. In XZ5, spike length, decreased only in D+S treatment; it remained unchanged in separate drought and salinity stress treatment. The grains per spike were significantly reduced in CM72 and XZ16; this component remained unaffected in XZ5 under all treatment compared to control. The three stress treatments significantly reduced percentage of grain-setting in CM72, while XZ16 and XZ5 remained unaffected (Table 1). 

**Table 1:** Effects of alone and combined stresses of drought and salinity during anthesis on yield and yield components of three barley genotype.

Treatment	Spike length (cm)	Filled grains per spike	Unfilled grains per spike	Rate of filled grains per spike (%)	1000 grain weight (g)	Grain yield per plant (g)
	**CM72**					
Control	5.83 a	31.67 a	5.00 b	86.37 a	37.95 a	2.08 a
Drought	3.57 d	14.67 d	11.68 a	55.67 d	26.97 c	0.76 d
	(-38.8)	(-53.7)	(+133.6)	(-35.5)	(-28.9)	(-63.5)
Salinity	5.33 b	28.02 b	8.01 ab	77.77 b	33.11 b	1.48 b
	(-8.57)	(-11.5)	(+60.2)	(-9.9)	(-12.8)	(-28.8)
D+S	4.51 c	20.01 c	10.02 a	66.63 c	24.54 d	1.06 c
	(-22.6)	(-36.8)	(+100.4)	(-22.8)	(-35.3)	(-49.1)
	**XZ16**					
Control	5.77 a	38.33 a	3.00 a	92.74 a	33.84 a	1.94 a
Drought	4.27 c	28.00 b	4.00 a	87.50 a	27.28 c	0.81 d
	(-25.9)	(-26.9)	(+33.3)	(-5.6)	(-19.4)	(-58.2)
Salinity	4.96 b	40.33 a	3.33 a	92.37 a	30.23 b	1.65 b
	(-14.1)	(+5.2)	(+11.0)	(-0.4)	(-10.7)	(-14.9)
D+S	4.17 c	30.67 b	4.00 a	88.46 a	24.03 d	0.99 c
	(-27.7)	(-19.9)	(+33.3)	(-4.6)	(-28.9)	(-48.9)
	**XZ5**					
Control	9.43 a	75.00 a	5.00 a	93.75 a	30.59 a	2.02 a
Drought	8.16 ab	57.66 a	10.00 a	85.22 a	24.26 b	1.15 c
	(-13.5)	(-23.1)	(+100)	(-9.1)	(-20.7)	(-43.1)
Salinity	8.53 ab	69.00 a	7.43 a	90.28 a	26.97 b	1.60 b
	(-9.5)	(-8.0)	(+48.6)	(-3.7)	(-11.8)	(-20.8)
D+S	7.83 b	60.33 a	9.67 a	86.18 a	22.20 c	1.16 c
	(-16.9)	(-19.6)	(+93.4)	(-8.1)	(-27.4)	(-42.6)

Different letters indicate significant differences (P<0.05) among three genotypes within each treatment (n=4). Values in parenthesis are expressed as a decreased (-)/increased (+) percentage of the control

During anthesis, barley plants that were subjected to drought and/or salinity exhibited significant decreases in grain yield and 1000-grain weight, with XZ5 showing the smallest affected. Although CM72 showed the highest grain yield and 1000-grain weight under control conditions, it also showed the most substantial changes during stress condition than in XZ16 and XZ5. For example, when averaged over the three treatments, a significant reduction was seen in grain yield/ 1000-grain weight in all genotypes (Table 1). Decreases in grain yield/ 1000-grain weight were 35.4%/ 19.9% in XZ5, 40.7%/ 19.6% in XZ16 and 47.1%/ 25.6% in CM72, respectively, as compared with controls. 

### Na^+^ and K^+^ concentrations in plant tissues

Both salinity and D+S treatments caused signiﬁcant (P<0.05) increases in Na^+^ concentration and Na^+^/K^+^ ratio in roots, stems and leaves in all genotypes, compared with controls (Table 2). Moreover, a preferential accumulation of Na^+^ in shoots (stem and leaf) rather than in roots was observed under salinity stress. In plants subjected to salinity and D+S treatment, shoot Na^+^ concentration increased on average by 86.2% in XZ5, 70.6% in XZ16 and 114.1% in CM72; root Na^+^ concentration increased by 92.9%, 328.5% and 132.3%, respectively, compared with their respective controls. Under drought stress alone, root Na^+^ concentration increased in the order XZ16 > XZ5 > CM72 (by 221.4%, 126.7% and 31.3%, respectively); no significant difference was observed in stems. Leaf Na^+^ concentration increased in CM72 and decreased in XZ5; however, no change was observed in XZ16 under drought stress alone.

**Table 2:** Root, shoot and leaf Na^+^ and K^+^ concentrations and Na^+^/K^+^ ratio of three barley genotypes during anthesis stage, exposure to alone and combined stresses of drought (4% soil moisture) and salinity.

Treatment	Na^+^ concentration (mg g^-1^ DW)			K^+^ concentration (mg g^-1^ DW)			Na^+^/K^+^ Ratio		
	Root	Leaf	Stem	Root	Leaf	Stem	Root	Leaf	Stem
	**CM72**								
Control	0.48 c	10.81 c	8.61 b	1.07 a	9.73 b	9.49 b	0.44 d	1.12 b	0.92 c
Drought	0.63 b	13.97 b	8.38 b	1.06 a	12.24 a	10.82 a	0.59 c	1.14 b	0.77 d
	(+31.3)	(+29.2)	(-2.6)	(-0.9)	(+25.8)	(+14.0)	(+34.1)	(+1.7)	(-16.3)
Salinity	0.59 bc	22.75 a	18.31a	0.87 b	8.61 c	9.30 b	0.69 b	2.67 a	1.97 b
	(+22.9)	(+110.4)	(+112.6)	(-18.7)	(+11.5)	(-2.0)	(+56.8)	(+138.4)	(+114.1)
D+S	1.64 a	21.09 a	20.52 a	0.94 b	9.19 c	5.42 c	1.74 a	2.31 a	3.76 a
	(+241.6)	(+95.1)	(+138.3)	(-12.1)	(-5.5)	(-42.9)	(+295.5)	(+106.3)	(+308.9)
	**XZ16**								
Control	0.28 c	14.90 b	10.45 b	0.90 c	9.99 b	8.34 b	0.31 c	1.50 bc	1.26 c
Drought	0.90 b	16.27 b	9.63 b	1.15 a	12.61 a	10.95 a	0.78 b	1.29 c	0.88 d
	(+221.4)	(+9.2)	(-7.8)	(+27.8)	(+26.2)	(+31.3)	(+151.6)	(-14.0)	(-30.2)
Salinity	0.86 b	20.65 a	20.30 a	1.09 b	10.54 b	8.74 b	0.79 b	1.98 a	2.33 b
	(+207.1)	(+38.6)	(+94.3)	(+21.1)	(+5.5)	(+4.8)	(-154.8)	(+32.0)	(+84.9)
D+S	1.54 a	20.12 a	22.42 a	1.08 b	11.85 a	8.04 b	1.45 a	1.71 b	2.83 a
	(+450.0)	(+35.0)	(+114.5)	(+20.0)	(+18.6)	(-3.6)	(+367.7)	(+14.0)	(+124.6)
	**XZ5**								
Control	0.56 c	18.15 b	9.76 b	1.25 b	9.66 c	8.16 b	0.45 d	1.90 bc	1.20 b
Drought	1.27 a	13.80 c	10.28 b	1.71 a	11.75 b	11.51 a	0.74 c	1.18 c	0.90 c
	(+126.7)	(-23.1)	(+5.3)	(+36.8)	(+21.6)	(+41.1)	(+64.4)	(-37.9)	(-25.0)
Salinity	0.98 b	26.51 a	22.12 a	1.01 c	11.71 b	7.20 b	0.98 b	2.27 a	3.07 a
	(+75.0)	(+46.1)	(+126.63)	(-19.2)	(+21.22)	(-11.8)	(+117.7)	(+19.47)	(+155.8)
D+S	1.18 ab	23.69 a	23.57 a	0.95 c	13.40 a	7.48 b	1.30 a	1.78 b	3.16 a
	(+110.7)	(+30.5)	(+141.5)	(-24.0)	(+38.7)	(-8.3)	(+188.9)	(-6.31)	(+163.3)

Different letters indicate significant differences (P<0.05) among three genotypes within each treatment (n=4). Values in parenthesis are expressed as a decreased (-)/increased (+) percentage of the control.

**Figure 2:**
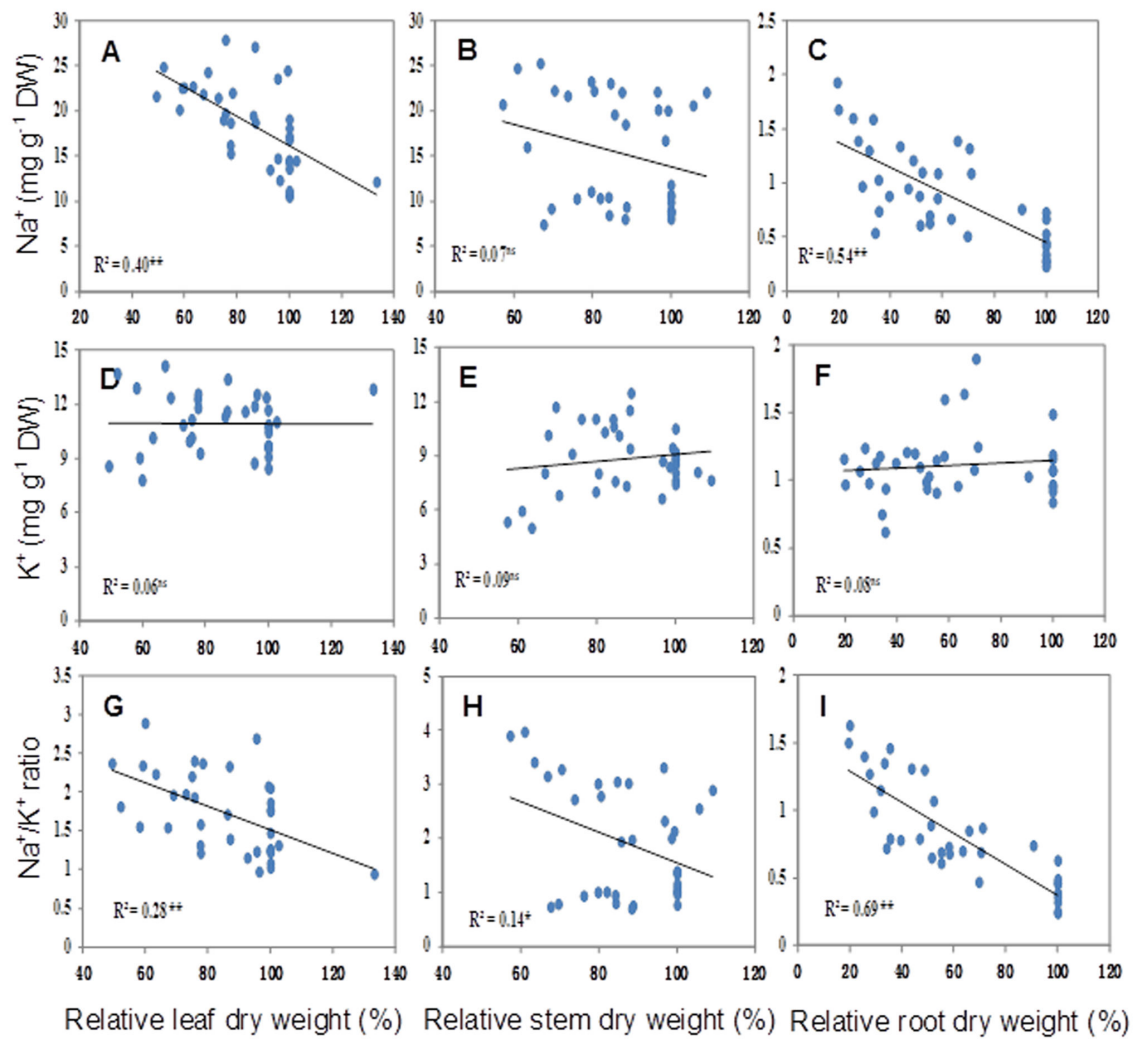
The correlation between relative dry weight and ionic concentration. The correlation between Na^+^, K^+^ concentration and Na^+^/K^+^ ratios and relative leaf (A, D and G), stem (B, E and H) and root (C, F and I) dry weight based on three genotypes: CM72, XZ16 and XZ5. ns’ not significant, * and ** indicate significant at 0.05 and 0.01, respectively.

A significant (P<0.05) decrease in the K^+^ concentration was detected in roots of XZ5 and CM72 under salinity alone and D+S stresses. An increase in K^+^ concentration was found in XZ16 under salinity and D+S treatments when compared with controls. The leaf and stem K^+^ concentrations decreased in CM72; but leaf K^+^ increased in XZ5 and XZ16, whereas no significant difference was observed in the stems of these genotypes. Under D+S treatment, both XZ5 and XZ16 exhibited decreases in average shoot and root Na^+^/K^+^ ratios of 20.1% and 23.3% respectively, compared to CM72 (Table 2). The Na^+^ concentrations in leaves and roots (Figure 2A and C) were found to be significantly correlated with the relative dry weight; no correlation was observed between stem Na^+^ concentration and the relative dry weight (Figure 2B). However, a significant correlation was obtained between leaf, stem and root Na^+^ /K^+^ ratios and their respective relative dry weight (Figure 2G, H and I).

### Photosynthetic parameters and chlorophyll content

Effects of salinity and drought stress either alone or combined during anthesis on photosynthetic parameters and cholorophyll content in flag leaves are summarized in Table S2. Drought, salinity and D+S treatments drastically inhibited net photosynthetic rate (Pn) and stomatal conductance (*gs*). Both drought and D+S treatments induced greater inhibitory effect than salinity stress alone. Among the three genotypes studies, the highest Pn and *gs* values were recorded in XZ5 in control, drought, salinity and D+S treatments; these measurement were significantly higher than those seen XZ16 and CM72. The Ci values declined in all genotypes under the three stress treatments. Greater Ci values were observed in the wild genotypes XZ5 and XZ16 relative to CM72 under drought and salinity combined stress. All stress treatments significantly reduced the transpiration rate (Tr) in all genotypes compared to their controls. However, the reduction in Tr was more pronounced in drought and D+S treatments than in salinity stress alone. Among the genotypes, Tr was considerably greater in CM72, than XZ16 and XZ5 under drought alone and D+S stresses. 

Compared with controls, chlorophyll content (Chl a, Chl b) in all the genotypes was significantly (P<0.05) reduced under stress (Table S2). Considering the level of Chl a, Chl b and carotenoids, the genotypes varied in response to different stress treatments. Salinity stress had no effect on Chl a, Chl b and carotenoids contents in XZ16, while these contents were significantly reduced in CM72 and XZ5. Under drought and D+S treatments, a similar reduction in Chl a and carotenoid contents was seen in all genotypes. However, reduction in Chl b content was more pronounced in D+S treatments compared to drought treatment (Table S2). Among the genotypes studied, chlorophyll contents were least affected in XZ16 compared with CM72 and XZ5 under all treatments. 

### Antioxidant enzyme activities

**Figure 3:**
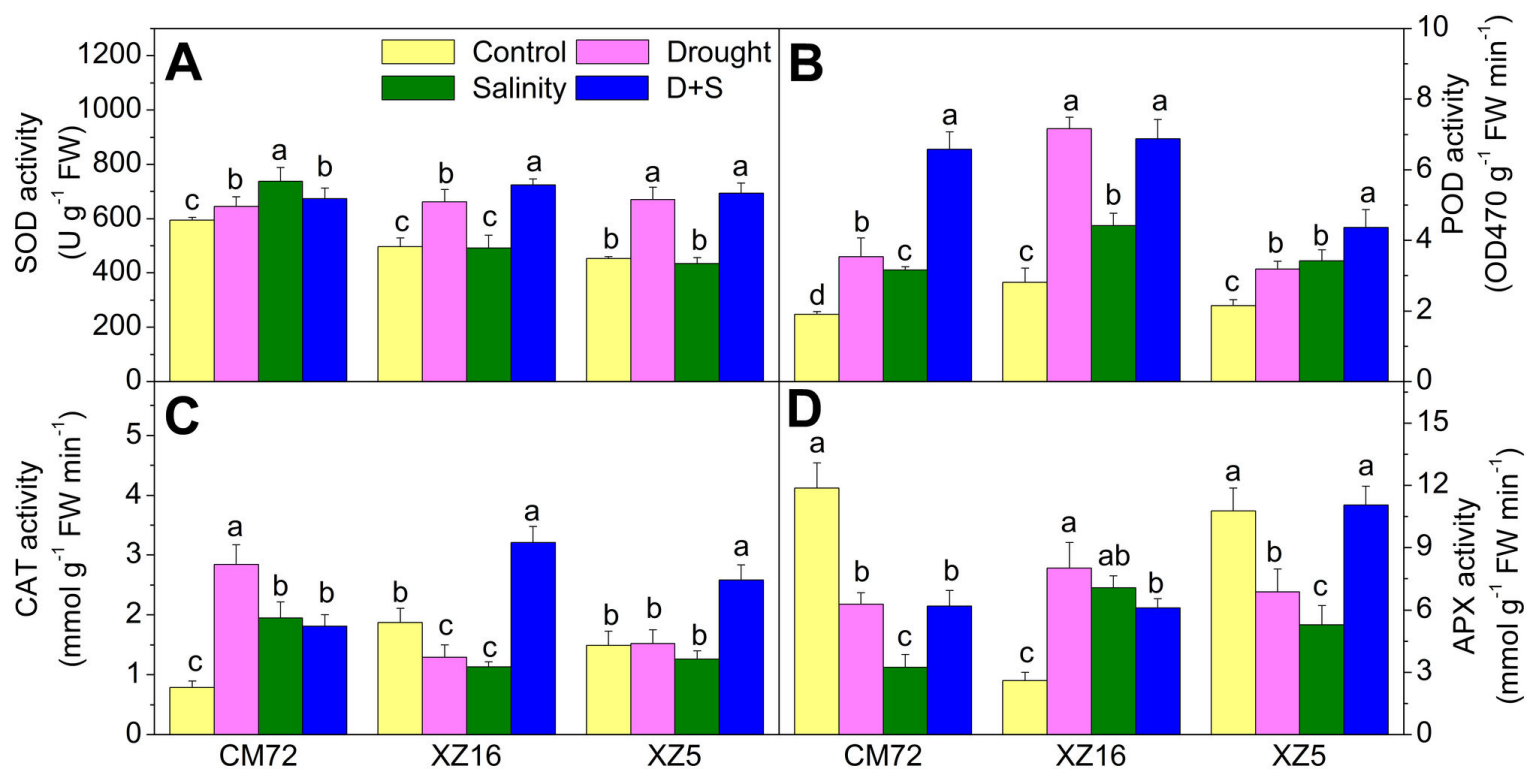
The antioxidant enzyme activities in cultivated and Tibetan wild barley genotypes. Barley plants were exposed to alone and combined stresses of drought and salinity on superoxide dismutase (SOD, A) , peroxidase (POD, B), catalase (CAT, C) and ascorbate peroxidase (APX, D) in flag leaves of three barley genotypes during anthesis stage at 4% soil moisture level. Error bars represent SD values (n=4). Different letters indicate significant differences (P<0.05) among the treatments within each genotype. Control, drought, salinity and D+S correspond to normal growth condition without drought and salinity stress, drought stress (D, seedlings were subjected to drought stress for 20 d during anthesis by withholding irrigation until the soil moisture content (SMC) reduced to 4%), salinity stress (S, 1 L 200 mM NaCl solution was added per pot at anthesis stage and then kept humid throughout the experimental period), combined stresses of drought and salinity (D+S, 1 L 200 mM NaCl solution was added per pot at anthesis stage, then subjected to drought stress until SMC reduced to 4%), respectively.

Antioxidant responses in Tibetan wild and cultivated barley genotypes to drought, salinity or D+S treatments are presented in Figure 3 and Table S3. The SOD activity in flag leaves significantly (P<0.05) increased in all genotypes under drought and D+S and in CM72 under salinity stress alone; however, no significant effect of salinity stress was observed in XZ5 and XZ16 compared with their controls (Figure 3A and Table S3). POD activity increased under drought, salinity alone and D+S treatments in all genotypes. The highest increases in POD activity were seen in XZ16 under drought and D+S treatment, and in CM72 under D+S treatment (Figure 3B and Table S3). CAT activity increased in CM72 and decreased in XZ16; no significant difference was seen in XZ5 under drought and salinity stress alone. However, in D+S treatment, CAT activities during anthesis at a soil moisture level of 4% were higher in Tibetan wild type genotypes than in CM72 (Figure 3C and Table S3). Compared to controls, APX activity decreased in CM72 and increased in XZ16 under the three stress treatments. In XZ5, APX activity decreased under drought and salinity stress separately, while no significant difference was observed under D+S treatment (Figure 3D and Table S3).

### Lipid peroxidation (MDA) contents and cell membrane stability index (CMSI)

**Figure 4:**
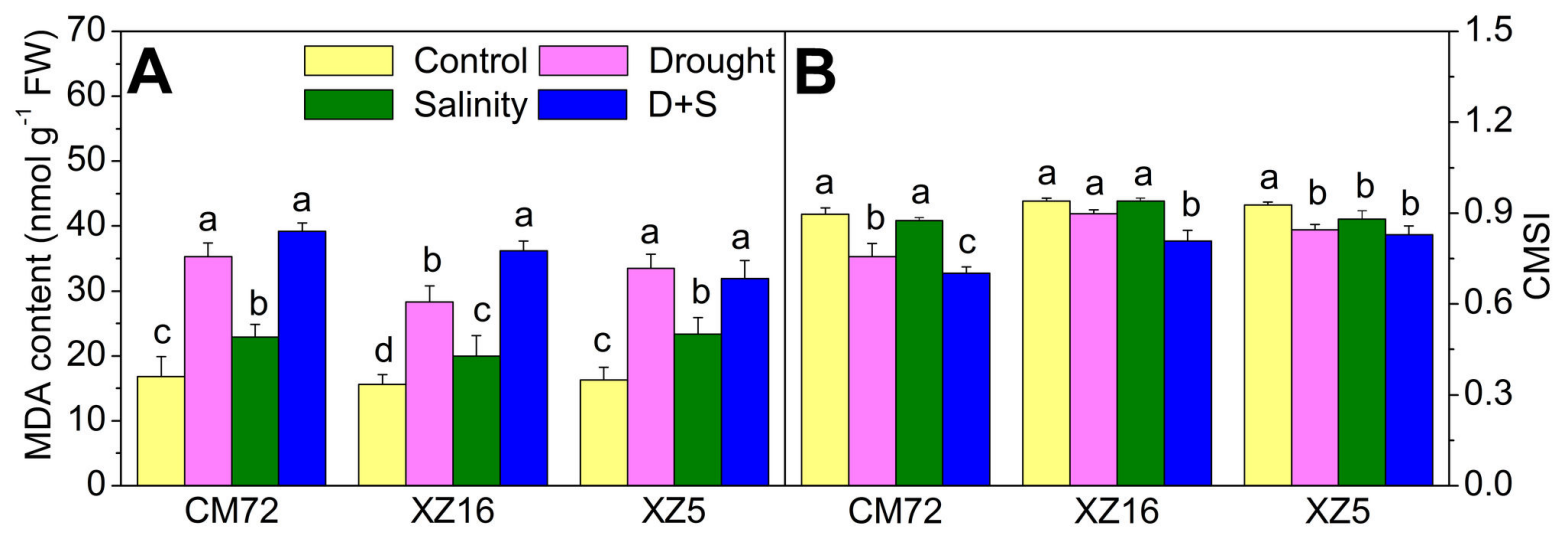
MDA content and CMSI in flag leaves of Tibetan wild and cultivated barley genotypes. Barley plants were exposed to drought, salinity alone and combined stresses during anthesis stage at 4% soil moisture level. Error bars represent SD values (n=4). Different letters indicate significant differences (P<0.05) among the treatments within each genotype. CMSI, cell membrane stability index; MDA, malondialdehyde;.

Plants exposure to drought, salinity and D+S stresses induced a treatment- dependent but genotype-independent marked increase in MDA content in flag leaves (Figure 4A and Table S3); greater accumulation was seen under drought and D+S treatments compared to salinity stress alone. CMSI changed slightly, when plants were exposed to drought and salinity stresses separately, relative to controls. Under D+S treatment, CMSI was markedly reduced in CM72 compared to XZ16 and XZ5. For example, the CMSI under D+S treatment decreased by 10.4%, 14.1% and 25.7% in XZ5, XZ16 and CM72, respectively, compared with their respective controls (Figure 4B Table S3).

### Protease activity and accumulation of glycine-betaine, soluble sugars and soluble protein

**Figure 5:**
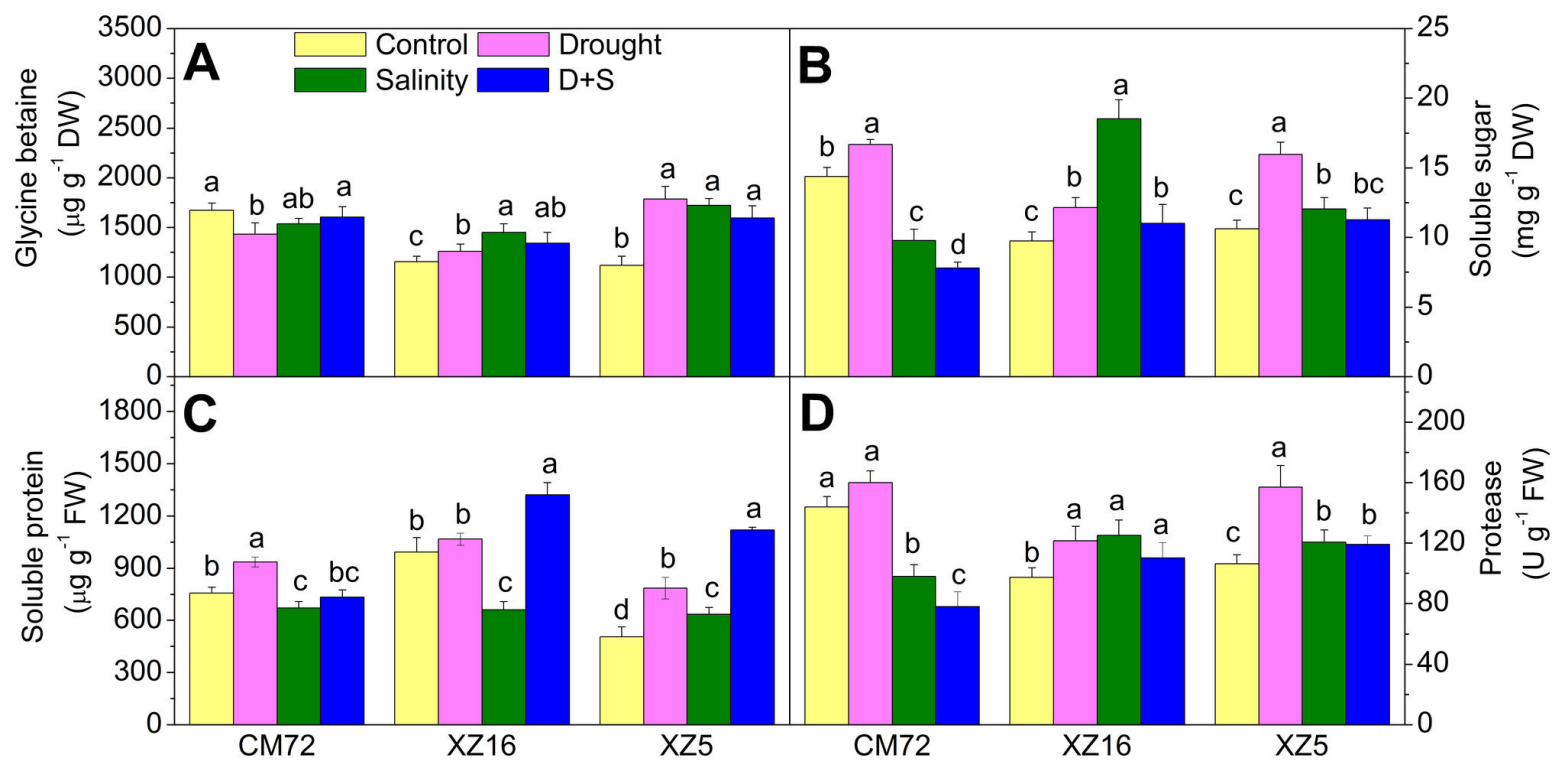
Glycine-betaine, soluble sugar, soluble protein and protease activity in flag leaves of three barley genotypes. Tibetan wild and cultivated barley plants were exposed to drought, salinity alone and combined stresses during anthesis stage at 4% soil moisture level. Error bars represent SD values (n=4). Different letters indicate significant differences (P<0.05) among the treatments within each genotype.

As shown in Figure 5A and Table S3 glycine-betaine (GB) content increased signiﬁcantly (P<0.05) in XZ5 and XZ16 (with a larger increase in XZ5) under all treatments. The GB content decreased in CM72 under drought stress alone; while no difference was seen in these genotypes under salinity alone and D+S treatment compared to the control plants. A greater increase in the soluble sugar content was observed in XZ16 compared to XZ5 under all treatments relative to controls. Soluble sugar content decreased in CM72 under salinity and D+S treatments and increased under drought (Figure 5B and Table S3). Soluble protein content also increased in flag leaves of XZ5 under all treatments, while in the case of XZ16 and CM72, an increase in soluble protein content was only observed under D+S treatment and drought treatment, respectively. Compared to the other genotypes, the protein level in XZ5 increased the fastest, and the magnitude of this increase was also the greatest under all treatments (Figure 5C and Table S3). Protease activity in flag leaves varies among genotypes in control and under different stress conditions. Under control conditions, protease activity was higher in CM72 compared to XZ5 and XZ16. However, under salinity alone and D+S treatments, protease activity increased signiﬁcantly (P<0.05) in XZ16 and XZ5 and decreased in CM72, compared to controls (Figure 5D and Table S3). 

### Non-enzymatic antioxidant and total phenol contents

**Figure 6:**
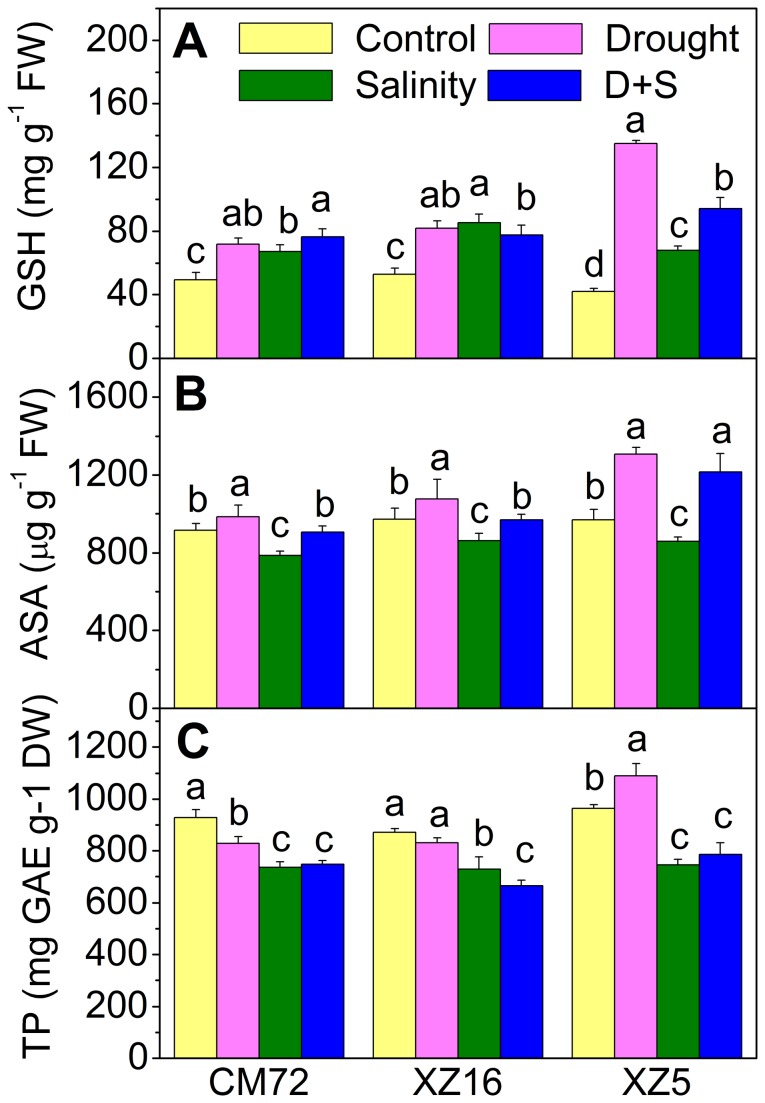
Reduced glutathione (GSH), reduced ascorbate (ASA) and total phenol (TP) contents in flag leaves of barley genotypes. Tibetan wild and cultivated barley plants were exposed to drought, salinity alone and combined stresses during anthesis stage at 4% soil moisture level. Error bars represent SD values (n=4). Different letters indicate significant differences (P<0.05) among the treatments within each genotype.

The contents of reduced glutathione (GSH), ascorbate (ASA) and total phenol (TP) contents under drought and salinity stress alone or in combination are shown in Figure 6 and Table S4. The GSH content was signiﬁcantly increased under all treatments in all genotypes compared to controls. Among the genotypes tested, the highest GSH content was seen in XZ5 under drought alone and D+S treatments. For example, GSH content under drought, salinity and D+S treatments increased by 222.1%, 61.6% and 124.7% in XZ5; 54.3%, 61.3% and 46.7% in XZ16 and 45.3%, 35.8% and 54.4% in CM72, respectively, compared with those of controls (Figure 6A and Table S4). ASA content decreased in all genotypes under salinity treatment. However, drought stress alone increased ASA content in all genotypes (c.f. increased by 34.7%, 10.8% and 7.7% in XZ5, XZ16 and CM72, respectively). Under D+S treatment, ASA content increased in XZ5 and remained unchanged in XZ16 and CM72 (Figure 6B and Table S4). Under drought conditions, total phenol content increased (P<0.05) only in XZ5; total phenol content decreased in CM72 and remained unaffected in XZ16 under drought condition, relative to controls. Salinity and D+S treatments led to a decrease in total phenol content in all genotypes during anthesis compared with their controls (Figure 6C and Table S4).

### Activity of ATPase in flag leaves

**Figure 7:**
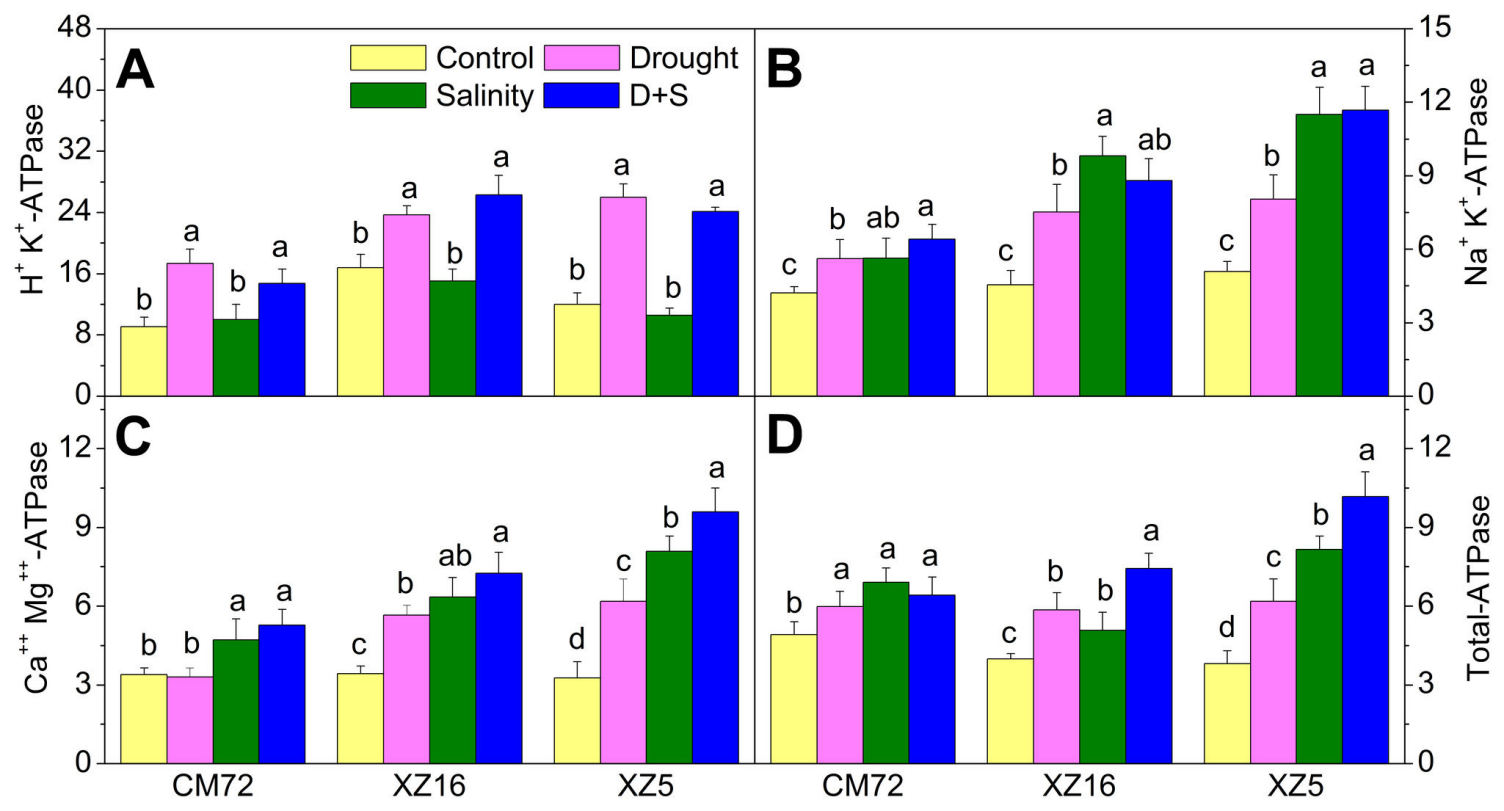
ATPase activity (µmol Pi mg^−1^ protein h^−1^) in flag leaves of three barley genotypes. Tibetan wild and cultivated barley plants were exposed to drought, salinity alone and combined stresses during anthesis stage at 4% soil moisture level. Error bars represent SD values (n=4). Different letters indicate significant differences (P<0.05) among the treatments within each genotype.

Compared to control, H^+^K^+^-ATPase activity in flag leaves signiﬁcantly increased under drought alone and D+S stresses for all genotypes, whereas no significant difference was detected under salinity stress (Figure 7A). Activity of Na^+^K^+^-, Ca^++^Mg^++^- and total-ATPases significantly increased under all treatments in all genotypes. Only activity of Ca^++^Mg^++^-ATPase in CM72 remained unchanged under drought stress alone, relative to controls. Under all stress treatments, the average percent increases in activities of Na^+^K^+^-, Ca^++^Mg^++^- and total-ATPases between the three genotypes in descending order were as follows: XZ5 > XZ16 > CM72 (Figure 7B-D and Table S4). 

## Discussion

Drought and salinity stresses impose major environmental threats to sustainable agriculture. The adverse impacts of these stresses are becoming more chronic in regions where saline water is used for irrigation [15]. Moreover, drought stress at the critical stages of anthesis and grain filling has a detrimental effect on grain quantity and quality traits [35-37]. The flag leaf of small cereal grain crops, such as barley and wheat, not only shields reproductive organs during development, but also serves as an important source of nutrients by substantially increasing in mass [38]. The physiological efficiency of a flag leaf seems to be the result of its structural rather than its enzymatic characteristics [39]. Therefore, flag leaves have a high rates of photosynthesis, nitrogen assimilation and dry matter per unit area [39]. The present study was carried out to monitor the effects of drought and salinity stress both alone and combined in flag leaves of barley at anthesis stage. This investigation will not only strengthen the understanding of stress tolerance mechanisms but will also provide a broader range for the degree of tolerance in all growth phases of Tibetan wild type barley. 

A reduction in plant growth was observed in our present work under drought and salinity stress, either alone or in combination. In plants under D+S treatment, associated with lowest stem, leaf and root dry weight values, resulting in decreased root/shoot dry weight ratio (Table S1). Genotype-specific growth inhibition under drought has been observed in barley and wheat [40]. Specifically, the leaf expansion rates were reduced under drought stress in the elite Israeli cultivar Seeva compared to wild barley [41]. Significant differences in spike morphology were detected between Tibetan wild and cultivated barley genotypes under drought conditions (Figure 1). Under drought stress, spikes were fully emerged in XZ5; however, spikes were almost covered by the flag leaf sheath in CM72. This result might be due to rolling in flag leaf sheath in response to inadequate moisture as a result of excessive transpiration loss and also negative peduncle extrusion. Under all stress treatments, the reduction in spike length was noticeably less in XZ5 than in XZ16 and CM72. The 1000-grain yield and the ﬁlled grains per spike measurements were correlated, which may explain the yield loss in cultivated barley compared to Tibetan wild barley under all treatments during the anthesis stage. The decline in yield decline was possibly associated with the reduction in spikelet fertility and grain filling (Table 1). These findings are supported by Kuixian et al. (42), who observed similar phenomena in rice genotypes. The yields of both CM72 and XZ16 were higher under D+S treatment than under drought treatment. This result could be associated with that both genotypes of CM72 and XZ16 are salinity tolerant, while sensitive to drought stress compared with XZ5. Plants subjected to drought and/or salinity stress undergo numerous physiological and metabolic changes. As to tolerant genotypes, salt stress induced numerous physiological and metabolic responses to cope with salt stress. Thus, D+S stress induced salt tolerance mechanisms to mitigate the damage in salinity tolerant CM72 and XZ16, but not in drought alone condition. A general decrease in photosynthetic rate is among the most common responses to water stress. In this study, higher net photosynthetic rate was observed in plants under D+S compared to drought treatment; the grain yield could be associated with these phenomena. The flag leaf has a high rate of photosynthesis, nitrogen assimilation and dry matter per unit area and, as a result, protects the reproductive organs and provides food for developing grains, which pending further study. 

It is generally accepted that genotypes that are able to sustain photosynthesis in flag leaf for a longer time tend to have greater yields. In our study, barley plants grown under drought, salinity and D+S treatments showed a marked reduction in chlorophyll content (Chl a, Chl b and carotenoids), accompanied by a sharp decrease in Pn, *gs*, and Tr. These results indicate that photosynthetic inhibition was caused by stomatal factors and by chlorophyll synthesis inhibition (Table S2). Farquhar and Sharkey [43] suggested that inhibition of photosynthesis was caused by stomatal factor (stomatal closure) or non-stomatal factors (impairments in metabolic processes) factors. A remarkable reduction in *gs* may be a consequence of stomatal closure caused by increased osmotic pressure in guard cells under D+S. This is an important stress avoidance mechanism in plants so that water is held via reduced transpiration. 

Tissue Na^+^ and K^+^ concentrations and the Na^+^/K^+^ ratios have been widely used as reliable indicators of salinity tolerance in barley [44]. An increase in Na^+^ ion concentration and a decrease in K^+^ ion uptake interrupt ionic balance as observed in most species exposed to salinity stress [13]. In our case, an increase in Na^+^ concentration and Na^+^/K^+^ ratios were observed in roots, stems and leaves in both wild and cultivated barley genotypes under salinity alone and D+S stress treatments compared with controls (Table 2). Salinity alone and D+S stresses induced a significant decrease in K^+^ concentration in roots of CM72 and XZ5 plants; however, K^+^ concentration in roots increased in XZ16. Interestingly, leaf K^+^ concentration in XZ5 and XZ16 increased under D+S treatment. This increase was greater in XZ5. Under D+S treatment, both XZ5 and XZ16 exhibited lower Na^+^/K^+^ ratios in leaves and stems than CM72 (Table 2). Thus, a low Na^+^/K^+^ ratio may be suitable for the metabolic processes occurring within the plants and may also be essential for plants to survive salt stress [45]. This low ratio may also indicates that the two Tibetan barley genotypes were more tolerant to salinity stress than CM72 [46]. These wild genotypes may contain elite alleles that can be used to improve salinity tolerance in barley.

Accumulation of MDA is an indicator of lipid peroxidation level, which reflects the extent of tolerance to abiotic stresses such as drought and salinity. It has been reported that cultivars with higher drought tolerance have lower MDA content when subjected to stress [36]. It was found that a salt-tolerant mulberry variety showed little change in MDA content under salt stress [47]. Our results clearly demonstrated that CM72 had a higher MDA content than XZ16 and XZ5 under drought and D+S treatments. This effect may be associated with a strong defense against oxidative stress in Tibetan wild barley genotypes compared to CM72. CMSI is also considered to be an indicator of stress tolerance in plants, including resistance to drought and salinity [48,49]. CMSI is often affected by lipid peroxidation caused by ROS under stress conditions [47], which results in the production of MDA, as shown in Figure 4A.

The essential role of antioxidative system to maintain a balance between the overproduction of reactive oxygen species (ROS) and their scavenging to keep ROS at a signaling level to reinstate metabolic homeostasis has already been established [50]. Plants can induce the expression of antioxidant enzymes, including SOD, POD, CAT and APX, to counteract oxidative damage. Tibetan wild barleys genotypes XZ16 and XZ5 showed signiﬁcant increases in SOD activities under drought and D+S treatments during anthesis period (Figure 2A). It is therefore assumed that Tibetan wild barley may be more efficient in scavenging ROS. Furthermore, APX activities under D+S treatment were strikingly different between CM72 and the two wild genotypes. In CM72, the APX activity significantly decreased by 47.9% under D+S treatment compared to control. In XZ16, APX activity significantly increased under D+S treatment. In XZ5, APX activity remained unchanged (Figure 2D and Table S3). These results suggest that XZ16 and XZ5 possess a high antioxidant capacity for APX to scavenge ROS, which was coupled with a higher antioxidant activity in the wild genotype during the vegetative stage in our previous study [16]. The highest ASA and GSH contents in flag leaves were observed in XZ5 under drought alone and D+ S stresses, which may offer protection against stress induced oxidative damage (Figure 6A, B). Total phenol content increased only in XZ5 under drought stress; this finding was in line with previous results [51]. Phenol compounds are involved in the protection against drought stress as conﬁrmed by the high TP values seen in XZ5 and participation of phenol compounds in antioxidant scavenging mechanism (Figure 6C).

 The increased GB content seen in Tibetan wild barley may protect on enzyme activity, including enzymes associated with sugar and amino acid metabolism [52,53], leading to the greater increases in soluble sugars seen in Tibetan wild barley compared to control plants. Thus, it is proposed that the two Tibetan wild barley genotypes may acquire more protection than *cv* CM72 in stress conditions as a result elevated GB contents and the greater osmotic protection of higher soluble sugar contents and antioxidant metabolism. Proteolysis usually serves to release amino acids for synthesis of stress induced/responsive proteins [54]. In the present study, soluble protein contents in the leaves were found to be elevated in Tibetan wild barley genotypes under drought alone and D+S treatment; this result seems to be a consequence of enhanced protease activity. An increase in protease activity may occur for proteolysis of proteins released as a result of membrane damage. These released amino acids may be used for increased synthesis of antioxidant enzymes, which are also proteinaceous in nature. The production of stress-related proteins such as dehydrins, in addition to the elevated levels of antioxidant enzymes under drought stress, may be a reason for increased protein level seen in leaves. Actually, stress responsive proteins are protective in nature. Dehydrins have been suggested to be stabilizers of nuclear or cytoplasmic macromolecules under drought stress conditions.

The H^+^K^+^-ATPases serve as the primary pump that generate a proton motive force driving the transport of other solutes, including Na^+^ and K^+^. In addition, the H^+^K^+^-ATPase is crucial for plant to adapt to stress. Our results show that drought alone and combined stress increased H^+^K^+^-ATPase activities in all genotypes. As a result, plants can better maintain of an electrochemical potential gradient to drive nutrient uptake under stress conditions. Increase in ATPase-mediated H^+^ translocation across the plasma membrane is a component of the plant cell response to salt imposition [55]. Dawood et al. [33] reported that Al caused depression of root ATPase activities, especially in H^+^-ATPase and Na^+^K^+^-ATPase; however, pre-treatment using NaHS resulted in up-regulation of both H^+^-ATPase and Na^+^K^+^-ATPase activities. Plasma membrane Na^+^K^+^-ATPase are ubiquitous P-type membrane transport proteins, which couple the energy derived from ATP hydrolysis to drive transport of solutes against their electrochemical gradients and are involved in transport of protons [56]. Zhang and Han [57] reported that enhanced UV-B radiation reduced Na^+^K^+^-ATPase activity in mitochondria, chloroplasts and cellular solutes of wheat seedlings, UV-B radiation induced damage to wheat seedlings in terms of activity of Na^+^K^+^-ATPase in various organelles can be repaired in part by He-Ne laser irradiation. The activities of Na^+^K^+^-, Ca^++^Mg^++^- and total-ATPase significantly increased in response to all treatments compared to controls (Figure 7). In descending order, the greatest increases in these enzyme activities were seen in XZ5, XZ16 and CM72. These increased activities may inhibit the activity of photophosphorylation and in turn result in the increased activity of ATPases to repair the damage caused by drought and salinity stress. Moreover, Ca^++^-ATPase located at the plasma membrane are involved in Ca^++^ extrusion into the apoplast. It has been proposed that augmented levels of apoplastic Ca^++^ limit the bypass flow by reducing the ‘leakiness’ of endodermal junctions [58] and thus would restrict Na^+^ translocation to the shoot.

Collectively, our results showed that, wild barley genotypes XZ5 and XZ16 are comparatively tolerant to D+S treatment compared to *cv* CM72, and this tolerance is associated with the lower shoot and root Na^+^/K^+^ ratios seen in these genotypes. In addition, enhanced contents of glycine-betaine, soluble sugars and soluble proteins and the increased protease activity seen in wild type barley genotypes may lead to a greater adaptation to D+S treatment compared to cultivated barley. Furthermore, increased contents of GSH and ASA and improved activity of antioxidant enzymes of SOD, CAT, POD and APX were beneficial in antagonizing oxidative stress, as indicated by the lower accumulation of MDA. In addition, the enhanced ATPase activity under D+S treatment could be a factor in the higher tolerance to D+S stress seen in XZ5 and XZ16. Our results also indicate that osmotic protection, antioxidant status, ATPase activity and lipid peroxidation in ﬂag leaves can be used as indices of D+S tolerance during the anthesis stage in Tibetan wild barley. We propose that these Tibetan barley genotypes could be of value in enhancement of combined stress tolerance in cultivated barley, which is important for further understanding of the mechanisms and identification of specific genes related to drought and salinity resistance and for future improvement of cultivated barley. 

## Supporting Information

Table S1 **Effects of alone and combined stresses of drought and salinity during anthesis on growth parameters in the three barley genotypes at 4% soil moisture level.**
(DOC)

Table S2 **Effects of alone and combined stresses of drought and salinity on photosynthetic parameters and chlorophyll contents in flag leaves of three barley genotype during anthesis at 4% soil moisture level.**
(DOC)

Table S3 **Effect of drought, salinity and D+S stress on antioxidant enzyme activity (SOD, POD, CAT and APX), MDA content, CMSI, glycine-betaine, soluble sugar, soluble protein and protease of wild and cultivated barley expressed as decreased (-)/increased (+) percentage of control.**
(DOC)

Table S4 **Effect of drought, salinity and D+S stress on reduced glutathione (GSH), reduced ascorbate (ASA), total phenol (TP) contents and ATPase activity (H^+^K^+^, Na^+^K^+^, Ca^++^Mg^++^ and total) of wild and cultivated barley expressed as decreased (-)/increased (+) percentage of control.**
(DOC)
