# Supplementary material for: Difference in Yield and Physiological Features in Response to Drought and Salinity Combined Stress during Anthesis in Tibetan Wild and Cultivated Barleys
Source: PLoS One. 2013 Oct 24;8(10):e77869. doi: 10.1371/journal.pone.0077869 (PMC3812012; doi:10.1371/journal.pone.0077869)
Supplement: Table S1 — Effects of alone and combined stresses of drought and salinity during anthesis on growth parameters in the three barley genotypes at 4% soil moisture level. (DOC) [file pone.0077869.s001.doc]

**Table S1.** Effects of alone and combined stresses of drought and salinity during anthesis on growth parameters in the three barley genotypes at 4% soil moisture level.

| Treatment | Plant height  (cm) | Leaf DW  (g plant^-1^) | Stem DW  (g plant^-1^) | Root DW  (g) | Root/shoot ratio |
| --- | --- | --- | --- | --- | --- |
|  | **CM72** |  |  |  |  |
| Control | 73.08 a | 1.31 a | 1.78 a | 0.74 a | 0.25 a |
| Drought | 50.33 c  (-31.1) | 1.19 b  (-9.2) | 1.38 c  (-22.4) | 0.60 b  (-18.9) | 0.22 b  (-12.0) |
| Salinity | 60.16 b  (-17.7) | 1.09 c  (-16.8) | 1.65 b  (-7.3) | 0.56 c  (-24.3) | 0.20 c  (-20.0) |
| D+S | 46.58 d  (-36.3) | 1.04 d  (-20.6) | 1.24 d  (-30.3) | 0.49 d  (-33.8) | 0.19 c  (-24.0) |
|  | **XZ16** |  |  |  |  |
| Control | 65.83 a | 1.49 a | 1.65 a | 0.90 a | 0.29 a |
| Drought | 53.75 c  (-18.4) | 1.35 b  (-7.5) | 1.48 b  (-10.3) | 0.79 b  (-12.2) | 0.26 b  (-10.3) |
| Salinity | 59.00 b  (-10.4) | 1.23 c  (-15.7) | 1.66 a  (+0.6) | 0.66 c  (-26.6) | 0.23 c  (-20.7) |
| D+S | 47.33 d  (-28.1) | 1.28 c  (-12.3) | 1.35 b  (-18.2) | 0.61 c  (-32.2) | 0.21 c  (-27.5) |
|  | **XZ5** |  |  |  |  |
| Control | 76.41 a | 1.88 a | 2.15 a | 0.99 a | 0.25 a |
| Drought | 65.58 c  (-14.2) | 1.78 a  (-5.3) | 1.88 b  (-12.5) | 0.79 b  (-20.2) | 0.23 b  (-8.0) |
| Salinity | 71.58 b  (-6.3) | 1.71 a  (-9.0) | 2.10 a  (-4.2) | 0.69 c  (-30.3) | 0.20 c  (-20.0) |
| D+S | 60.66 d  (-20.6) | 1.40 b  (-26.6) | 1.71 c  (-20.5) | 0.67 c  (-32.3) | 0.19 c  (-24.0) |

Data were means of four independent replications.

Different letters indicate significant differences (P<0.05) among the four treatments and refer to data of each genotype. Values in parenthesis are expressed as a decreased (-)/increased (+) percentage of the control.
