# Supplementary material for: Difference in Yield and Physiological Features in Response to Drought and Salinity Combined Stress during Anthesis in Tibetan Wild and Cultivated Barleys
Source: PLoS One. 2013 Oct 24;8(10):e77869. doi: 10.1371/journal.pone.0077869 (PMC3812012; doi:10.1371/journal.pone.0077869)
Supplement: Table S2 — Effects of alone and combined stresses of drought and salinity on photosynthetic parameters and chlorophyll contents in flag leaves of three barley genotype during anthesis at 4% soil moisture level. (DOC) [file pone.0077869.s002.doc]

**Table S2.** Effects of alone and combined stresses of drought and salinity on photosynthetic parameters and chlorophyll contents in flag leaves of three barley genotype during anthesis at 4% soil moisture level.

| Treatment | Pn  (μmol CO_2_ m^-2^ s^-1^) | Gs  (mol H_2_O m^-2^ s^-1^) | Ci  (μmol CO_2_ mol^-1^) | Tr  (mmol H_2_O m^-2^ s^-1^) | Chl a  (mgg^-1^ FW) | Chl b  (mgg^-1^ FW) | Carotenoids  (mgg^-1^ FW) |
| --- | --- | --- | --- | --- | --- | --- | --- |
|  | **CM72** |  |  |  |  |  |  |
| Control | 7.52 a | 0.055a | 243.9 a | 0.940 a | 6.84 a | 2.10 a | 1.87 a |
| Drought | 1.93 d  (-74.3) | 0.015 c  (-71.1) | 128.6 d  (-47.3) | 0.409 c  (-56.5) | 4.02 c  (-41.2) | 1.27 c  (-39.5) | 1.01 c  (-45.9) |
| Salinity | 5.96 b  (-20.7) | 0.043 b  (-17.3) | 155.4 c  (-36.3) | 0.867 b  (-7.8) | 5.06 b  (-26.1) | 1.76 b  (-16.2) | 1.58 b  (-15.5) |
| D+S | 2.99 c  (-60.2) | 0.019 c  (-65.5) | 205.7 b  (-15.7) | 0.456 c  (-51.5) | 3.98 c  (-41.8) | 0.91 d  (-56.7) | 0.98 c  (-47.6) |
|  | **XZ16** |  |  |  |  |  |  |
| Control | 6.81 a | 0.042 a | 284.1 a | 0.744 b | 5.53 a | 1.90 a | 1.52 a |
| Drought | 1.99 d  (-70.7) | 0.012 c  (-71.4) | 151.9 b  (-46.7) | 0.261 d  (-64.9) | 3.44 b  (-37.6) | 1.41 b  (-25.7) | 0.97 b  (-36.2) |
| Salinity | 5.69 b  (-9.8) | 0.044 a  (+18.9) | 168.7 b  (-40.78) | 0.987 a  (+32.7) | 5.07 a  (-8.3) | 1.70 a  (-10.5) | 1.38 a  (-9.2) |
| D+S | 3.05 c  (-55.6) | 0.016 b  (-56.8) | 121.8 c  (-57.2) | 0.350 c  (-52.9) | 4.04 b  (-26.9) | 1.12 c  (-41.1) | 1.01 b  (-33.6) |
|  | **XZ5** |  |  |  |  |  |  |
| Control | 7.80 a | 0.058 a | 238.1 a | 1.032 a | 7.61 a | 2.41 a | 2.10 a |
| Drought | 2.99 d  (-61.7) | 0.022 c  (-62.1) | 149.8 c  (-37.8) | 0.249 c  (-75.8) | 4.45 b  (-41.5) | 1.87 b  (-22.4) | 1.31 b  (-37.6) |
| Salinity | 7.03 b  (-9.8) | 0.062 a  (+7.6) | 172.0 b  (-27.7) | 0.957 a  (-7.2) | 4.91 b  (-35.5) | 2.08 b  (-13.7) | 1.41 b  (-32.8) |
| D+S | 4.08 c  (-47.6) | 0.028 b  (-56.7) | 131.6 d  (-44.7) | 0.315 b  (-69.4) | 4.49 b  (-40.9) | 1.08 c  (-55.1) | 1.29 b  (-38.6) |

Data were means of four independent replications.

Different letters indicate significant differences (P<0.05) among three genotypes within each treatment. Values in parenthesis are expressed as a decreased (-)/increased (+) percentage of the control.
