# Supplementary material for: Difference in Yield and Physiological Features in Response to Drought and Salinity Combined Stress during Anthesis in Tibetan Wild and Cultivated Barleys
Source: PLoS One. 2013 Oct 24;8(10):e77869. doi: 10.1371/journal.pone.0077869 (PMC3812012; doi:10.1371/journal.pone.0077869)
Supplement: Table S3 — Effect of drought, salinity and D+S stress on antioxidant enzyme activity (SOD, POD, CAT and APX), MDA content, CMSI, glycine-betaine, soluble sugar, soluble protein and protease of wild and cultivated barley expressed as decreased (-)/increased (+) percentage of control. (DOC) [file pone.0077869.s003.doc]

**Table S3.** Effect of drought, salinity and D+S stress on antioxidant enzyme activity (SOD, POD, CAT and APX), MDA content, CMSI, glycine-betaine, soluble sugar, soluble protein and protease of wild and cultivated barley expressed as decreased (-)/increased (+) percentage of control.

| Treatment | Antioxidant enzyme activity | | | | MDA | CMSI | Glycine betaine | Soluble sugar | Soluble protein | Protease |
| --- | --- | --- | --- | --- | --- | --- | --- | --- | --- | --- |
|  | SOD | POD | CAT | APX |  |  |  |  |  |  |
|  | **CM72** |  |  |  |  |  |  |  |  |  |
| Drought | +8.65 | +85.31 | +261.72 | -47.09 | +109.72 | -10.18 | -14.35 | +16.09 | +23.58 | +11.22 |
| Salinity | +24.30 | +65.28 | +148.59 | -72.70 | +36.24 | +0.89 | -8.23 | -31.91 | -11.18 | -31.91 |
| D+S | +13.41 | +244.92 | +130.33 | -47.87 | +133.31 | -25.67 | -6.01 | -45.77 | -3.25 | -45.78 |
|  | **XZ16** |  |  |  |  |  |  |  |  |  |
| Drought | +33.19 | +154.25 | -28.52 | +214.73 | +81.44 | -2.44 | +9.08 | +24.54 | +7.54 | +24.55 |
| Salinity | -0.85 | +56.77 | -37.51 | +49.78 | +28.25 | -0.106 | +25.26 | +90.01 | -33.50 | +28.47 |
| D+S | +45.90 | +154.29 | +77.47 | +8.14 | +131.78 | -14.05 | +16.15 | +13.09 | +33.14 | +13.09 |
|  | **XZ5** |  |  |  |  |  |  |  |  |  |
| Drought | +47.93 | +47.63 | +1.61 | -36.21 | +106.11 | -3.45 | +59.38 | +50.19 | +55.66 | +47.67 |
| Salinity | -3.94 | +58.56 | -15.76 | -50.87 | +43.70 | -6.18 | +53.53 | +13.51 | +25.92 | +13.51 |
| D+S | +53.19 | +102.52 | +73.13 | +2.67 | +96.42 | -10.41 | +42.41 | +6.01 | +121.70 | +12.28 |

Values are obtained from Fig.3 to 5.
