# Supplementary material for: Difference in Yield and Physiological Features in Response to Drought and Salinity Combined Stress during Anthesis in Tibetan Wild and Cultivated Barleys
Source: PLoS One. 2013 Oct 24;8(10):e77869. doi: 10.1371/journal.pone.0077869 (PMC3812012; doi:10.1371/journal.pone.0077869)
Supplement: Table S4 — Effect of drought, salinity and D+S stress on reduced glutathione (GSH), reduced ascorbate (ASA), total phenol (TP) contents and ATPase activity (H+K+, Na+K+, Ca++Mg++ and total) of wild and cultivated barley expressed as decreased (-)/increased (+) percentage of control. (DOC) [file pone.0077869.s004.doc]

**Table S4.** Effect of drought, salinity and D+S stress on reduced glutathione (GSH), reduced ascorbate (ASA), total phenol (TP) contents and ATPase activity (H^+^K^+^, Na^+^K^+^, Ca^++^Mg^++^ and total) of wild and cultivated barley expressed as decreased (-)/increased (+) percentage of control.

| Treatment | GSH | ASA | TP | ATPase activity | | | |
| --- | --- | --- | --- | --- | --- | --- | --- |
|  |  |  |  | H^+^K^+^ | Na^+^K^+^ | Ca^++^Mg^++^ | Total |
|  | **CM72** |  |  |  |  |  |  |
| Drought | +45.35 | +7.69 | -10.57 | +90.86 | +33.31 | -2.54 | +22.01 |
| Salinity | +35.80 | -14.22 | -20.52 | +10.40 | +33.85 | +39.48 | +40.73 |
| D+S | +54.39 | -0.94 | -19.24 | +61.88 | +52.31 | +56.03 | +30.73 |
|  | **XZ16** |  |  |  |  |  |  |
| Drought | +54.39 | +10.75 | -4.56 | +40.82 | +64.98 | +64.89 | +46.88 |
| Salinity | +61.38 | -11.45 | -16.34 | -10.50 | +115.36 | +84.84 | +27.38 |
| D+S | +46.71 | -0.40 | -23.72 | +56.41 | +94.13 | +110.89 | +86.74 |
|  | **XZ5** |  |  |  |  |  |  |
| Drought | +222.16 | +34.71 | +12.20 | +116.20 | +58.09 | +89.12 | +62.55 |
| Salinity | +61.65 | -11.32 | -22.65 | -11.98 | +126.22 | +146.72 | +114.44 |
| D+S | +124.76 | +25.21 | -18.41 | +100.42 | +129.40 | +193.13 | +167.27 |

Values are obtained from Fig.6 to 7.
